# A Multiply and Long‐Chain Branched Polyolefin with Low Density Polyethylene (LDPE)‐Like Properties Containing Two Types of Functional Groups

**DOI:** 10.1002/anie.202518150

**Published:** 2025-10-23

**Authors:** Christoph Unger, Christian Heber, Holger Schmalz, Christof Bauer, Winfried P. Kretschmer, Rhett Kempe

**Affiliations:** ^1^ Anorganische Chemie II–Katalysatordesign, Sustainable Chemistry Centre Universität Bayreuth Universitätsstraße 30 NW I Bayreuth D‐95440; ^2^ Macromolecular Chemistry University of Bayreuth Universitätsstraße 30 95447 Bayreuth Germany; ^3^ Bavarian Polymer Institute (BPI) University of Bayreuth Universitätsstraße 30 95447 Bayreuth Germany

**Keywords:** Circular economy, Closed‐loop recycling, Coordinative chain transfer polymerization, LDPE‐mimic, Polyethylene

## Abstract

Low‐density polyethylene (LDPE) is an important material or plastic, which, unfortunately, is synthesized under very harsh conditions and challenging to recycle. We report here on a highly flexible catalytic synthesis of multiply branched and long‐chain branched polyolefins under mild conditions. We combine coordinative chain transfer polymerization of ethylene and branched alpha‐olefins with ring‐opening metathesis polymerization and hydrogenation catalysis. Our plastic contains two types of functional groups: olefins and esters. The olefin functional groups can be used to enable closed‐loop recycling, and the ester groups permit faster decomposition than LDPE to potentially reduce the micro‐plastic formation or enable a second depolymerization pathway. Our material matches key properties of LDPE, such as the melting point, rheology and stress–strain behavior.

## Graphical abstract

The combination of coordinative chain transfer polymerization of ethylene and branched alpha‐olefins, ring‐opening metathesis polymerization and hydrogenation catalysis yield a highly flexible catalytic synthesis of multiply and long‐chain branched polyolefins under mild conditions with two types of functional groups.

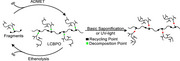

Polyethylene materials, such as low‐density polyethylene (LDPE), high‐density polyethylene, and linear low‐density polyethylene, are made in about 100 million ton scale annually, the highest mass of any plastic produced.^[^ ^1^, ^2^ ^]^ Of the three, LDPE is the material which has been known longest, discovered by Imperial Chemical Industries in the 1930s^[^ ^3^ ^]^ and still produced in a large scale and under harsh conditions (around 2000 bar and 200 °C) in a free‐radical process. The structure of LDPE is characterized by multiple branches and long‐chain branches which are difficult to synthesize with other methods.^[^ ^4^, ^5^, ^6^ ^]^ The molecular weight of the LDPE long‐chain branches should be higher than 800 g mol^−1^ to permit entanglement.^[^ ^7^, ^8^, ^9^ ^]^ An important characteristic resulting from that structure is shear thinning (shear flow), a reduction of viscosity with an increasing shear rate which is important for numerous applications, for instance (food) packing. Froese et al. reported very recently on a commercially viable solution process to control long‐chain branching in polyethylene.^[^ ^10^ ^]^ The key is a ladder‐like polyethylene material (Figure 1a) synthesized by a solution copolymerization of ethylene and alpha, omega‐dienes (less than 1 mol%) employing a catalyst that can grow two polymer chains per metal center simultaneously. The material introduced is still challenging to depolymerize and decomposes very slowly if uncontrolled release takes place, like all the classic polyethylene (PE) plastics. We reported an elongation/branching reaction of olefins and two ethylene molecules recently (Figure 1b).^[^ ^11^ ^]^ The elongation/branching reaction of 1‐hexene gives rise to 4‐ethyleoctene (4‐EO). The latter forms 2‐ethylhexyl branches if copolymerized with ethylene, which is proposed as the most common short‐chain branch structure in LDPE.^[^ ^12^ ^]^ In addition, we reported a coordinative chain‐transfer polymerization (CCTP) catalyst^[^ ^13^, ^14^, ^15^, ^16^, ^17^, ^18^ ^]^ transferring highly efficiently to the inexpensive chain transfer reagent triethylaluminium. This catalyst family permits the controlled and efficient polymerization of ethylene^[^ ^19^ ^]^ and the efficient and controlled copolymerization of ethylene and linear alpha‐olefins.^[^ ^6^ ^]^ We report here on the synthesis of differently functionalized and multiply branched and long‐chain branched polyolefin materials. Our polymers were synthesized under mild conditions, applying coordinative chain‐transfer polymerization and ring‐opening metathesis polymerization and hydrogenation catalysis. The functionalities introduced permit closed‐loop recycling (olefin) and accelerated decomposition or depolymerization (ester). Properties, such as the melting point and tensile strength, are adjustable and can match those of LDPE. In addition, PE‐like rheological properties are observed. Impressive progress in closed‐loop recyclable PE materials and polyolefin‐like materials has been reported in recent years with the focus on linear structures or functional groups that crosslink under processing conditions.^[^ ^6^, ^20^, ^21^, ^22^, ^23^, ^24^, ^25^, ^26^, ^27^, ^28^ ^]^ The PPWR (packing and packing waste regulation) of the EU is in place since February 2025 and requires packing materials to be recyclable till 2030 and recyclable in large scale till 2035.^[^ ^29^ ^]^

**Figure 1:**
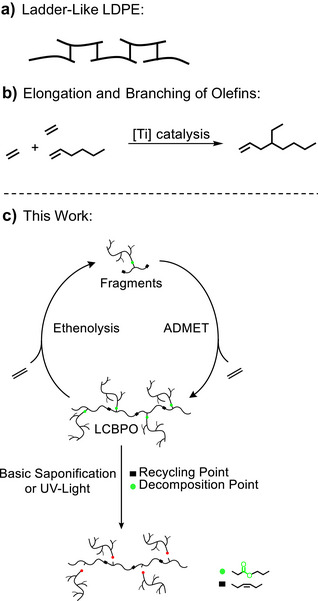
State of the art and the work presented here. a) Ladder‐like LDPE synthesized by catalytic copolymerization of ethylene and dienes under mild conditions in solution.^[^ ^10^ ^]^ b) Synthesis of branched α‐olefins via elongation and branching of olefins by two ethylene molecules.^[^ ^11^ ^]^ c) Multiply branched and long‐chain branched polyolefin (LCBPO) with two types of functional groups.

Firstly, we copolymerized ethylene with 4‐ethyl‐oct‐1‐ene (4‐EO) and aimed for a chain length between 1000 and 3 000 g mol^−1^ (Table 1).^[^ ^8^, ^9^ ^]^ The copolymers are characterized by narrow molecular weight distributions, which are typical for products synthesized by CCTP.^[^ ^19^ ^]^ We oxidized the polymer chains resting on aluminum after copolymerization and yielded multiple branched alcohols. The multiple branched alcohols (**P1**) are characterized by 2‐ethyl‐hexyl branches, which is a typical structure motive of classic LDPE materials.^[^ ^12^, ^27^ ^]^ The ^1^H nuclear magnetic resonance (NMR) analysis of the reference ethylene homopolymers for the chain length desired (Entry 1 and 3 of Table 1) indicates approximately 70%–74 % alcohol functionalization. We next functionalized the **P1** with norbornene units by esterification with the corresponding acid chloride (**P2**). The ^1^H NMR analysis of **P2** revealed ester groups and polymerizable cyclic olefinic units (Figure 2a). A quantitative conversion of the alcohol groups to the ester‐linked norbornene units was observed (Figure 2b). We subsequently became interested in the copolymerization of **P2** with cis‐cyclooctene (COE) to form a long‐chain branched polyolefin material (**P3**). We copolymerized **P2** with COE by using the Hoveyda–Grubbs catalyst of the second generation [HG II]^[^ ^30^ ^]^ and stopped the reaction by adding an excess of ethylvinylether (EVE). The results are listed in Table 2.

**Table 1:** (Co)polymerization of ethylene(/4‐EO) for the synthesis of multiply branched alcohols **P1**.^a)^

Entry	*n*_TEA_ [mmol]	*V*_4‐EO_ [mL]	*V*_eth_ [L_n_]	*M*_n_ [g mol^−1^]	Đ	activity^b)^ [$\frac{kg_{\mathrm{PE}}}{\mathrm{mo}l_{\mathrm{cat}}\cdot h\cdot \mathrm{bar}}$]	average 4EO u/c^c)^
1^d)^	2.5	/	8	1 360	1.5	13 800	/
2(**P1A**)	2.5	50	8	1 640	1.5	6 300	1.7
3	2.0	/	16	3 530	1.3	21 800	/
4(**P1B**)	2.0	50	16	3 440	1.6	2 400	3.2

^a)^ Reactants and conditions: *V*_toluene/4‐EO_ = 180 mL, *T* = 80 °C (70 °C for entry 1 and 2), *p*_ethylene_ = 3.0 bar (2.0 bar entry 1), *n* **_[Zr]_** = 1 µmol (2 µmol for copolymerizations), *n*_d‐MAO_  =  500 eq to [Zr], *p*_O2_  = 3 bar, *t*_oxidation_ = 3 h.

^b)^ Activity of the catalyst is based on the reaction time to consume the amount of ethylene desired.

^c)^ Incorporation of the average 4‐EO units per chain (u/c) is calculated by gel permeation chromatography (M_n_) and ^13^C NMR (Supporting Information).

^d)^ Data were taken from literature.^[^ ^6^ ^]^

**Figure 2:**
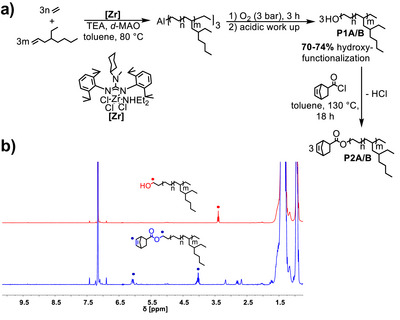
Synthesis of the multiply branched alcohols **P1** and its functionalization to **P2**. a) Synthesis of **P1** and **P2**. b) ^1^H NMR stack of **P1** (red) and **P2** (blue).

**Table 2:** Results of the (co)polymerization of **P2** with cyclooctene (COE) for the synthesis of **P3**.^a)^

Entry	COE [g]	P2 [g]	P2 [Type]	*M*_n_ [g mol^−1^]	Đ
5	2.0	/	/	670	1.5
6(**P3A**)	2.0	0.5	**A**	1 520	2.8
7(**P3B**)	2.0	0.5	**B**	2 670	3.6
8*(**P3Bs**)	2.0	0.5	**B**	1 360	3.8

^a)^ Reactants and conditions: *V*_toluene_ = 40 mL, *T* = 45–55 °C, *n* **_[HG II]_** = 1.81 µmol, *V*_EVE_  =  1 mL, *p*_O2_  = 3 bar, reaction time = 4 h. *COE was added stepwise over 30 min.

The thermal and mechanical properties of **P3** were compared with polycyclooctene (PCOE) synthesized under the same reaction conditions and commercial LDPE (Figure 3). We obtained two melting points (∼50 °C, ∼105 °C) and two recrystallization points/areas (∼90 °C, −30–40 °C) for **P3A** and **B** (Figure 3b). The thermogravimetric analysis curves of **P3A** and **B** and LDPE show similar behavior, in contrast to PCOE, that starts decomposition earlier (Figure 3b). The copolymer **P3B** showed a high elongation at a break of up to 1400%, with a slightly different tensile strength to LDPE. We carried out a partial hydrogenation of the unsaturated polyolefin backbone to obtain a single melting point (**P4**). The remaining double bonds can be used as recycling points with depolymerization and repolymerization using olefin metathesis permitting closed‐loop recycling. We used a reusable heterogeneous catalyst consisting of Pt nanoparticles (mean particle size of 2.2 nm) on a N and Si‐doped carbon support (N‐SiCN)^[^ ^31^ ^]^ for the hydrogenation reaction. A degree of hydrogenation of about 80% (analyzed by NMR spectroscopy, Figure 4a) was reached after 15–18 h applying 40 bar H_2_ pressure (Table S4). The NMR analysis of **P4** also revealed intact ester groups beside the degree of hydrogenation. A single melting point in the range between 100 and 115 °C was obtained after the hydrogenation. The mechanical properties of **P4B** can match those of a commercially available LDPE (Figure 4c, green curve). The rheological properties of our partially unsaturated polyolefin material **P4B** were investigated next. Frequency sweeps in the melt at different temperatures were conducted to demonstrate the shear thinning behavior. The rheological properties of our material were compared with commercial LDPE (Figures 5a and S48–S49). We observed a similar frequency dependence of the dynamic moduli, with a crossover of the storage (*G* ^′^) and loss (*G* ^″^) modulus in a comparable frequency range. In addition, despite the overall higher viscosity of our material, the shear thinning behavior is comparable to that of LDPE.

**Figure 3:**
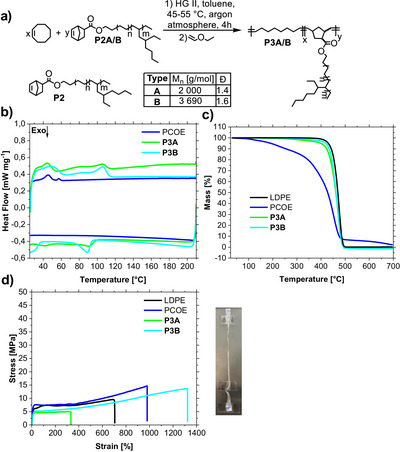
a) Copolymerization reaction of **P2** with COE to synthesize **P3**. b) Dynamic scanning calorimetry (second heating and cooling cure) of **P3** in comparison to polycyclooctene (PCOE). c) Thermogravimetric analysis curve of **P3** in comparison to PCOE and commercial LDPE. d) Representative stress–strain curves for **P3**, PCOE and LDPE (measured with a ramp speed of 10 mm min^−1^).

**Figure 4:**
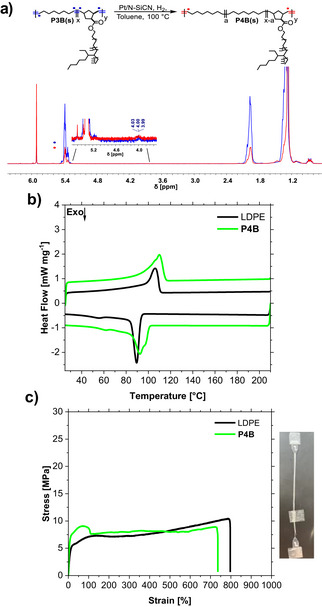
a) Hydrogenation reaction of **P3Bs** to **P4Bs**. b) DSC (second heating and cooling cure) of **P4B** in comparison to LDPE. c) Representative stress–strain curves for **P4B**, and LDPE (measured with a ramp speed of 5 mm min^−1^).

**Figure 5:**
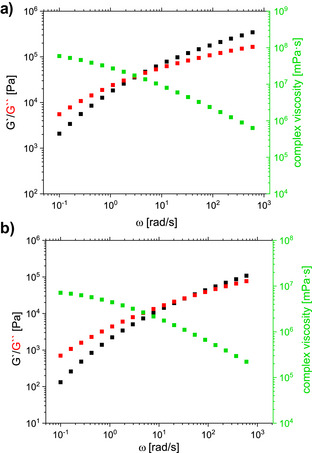
a) Frequency sweep in the melt of LCPBO (**P4B**) at 130 °C. b) Frequency sweep in the melt of commercial LDPE at 130 °C.

Depolymerization experiments were then conducted in which the polyolefin backbone is cleaved by an excess of ethylene to form telechelic α‐olefin fragments. The resulting telechelic polymer fragments were reconnected by acyclic diene metathesis experiments to demonstrate their repolymerization capability (Figure 6a). The recycling cycle was evaluated by NMR spectroscopy (Figure 6b). Olefin‐terminated fragments **P4Bs(depoly)** were yielded (blue spectra) by the olefin metathesis reaction of **P4Bs** with ethylene. Nearly quantitative conversion of these fragments to the recycled LCBPO **P4Bs2** (green spectrum) was observed by acyclic diene metathesis in a mixture of boiling hexane and toluene. Moreover, the molecular weight distributions showed the decomposition to lower molecular weights by ethenolysis (Figure 6c, blue curve). The molecular weight increases again (green curve) after the repolymerization step.

**Figure 6:**
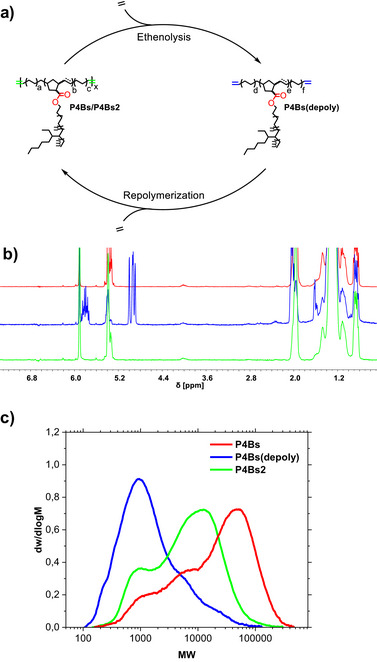
a) Closed‐loop recycling of **P4** by ethenolysis and acyclic diene metathesis. b) ^1^H‐NMR stack of the original LCBPO (**P4Bs**, red), its depolymerization by ethenolysis (blue) and the repolymerized LCBPO (green). c) Molecular weight distributions of the polymers **P4Bs**, **P4Bs(depoly)** and **P4Bs2**.

Finally, we tested **P4B** in UV light‐mediated decomposition reactions, evaluated the decomposition product by gel permeation chromatography and NMR spectroscopy, and compared it with commercial LDPE (Figure 7). Compression‐molded disc‐like samples were irradiated with UV light (wavelength 300–400 nm) in the presence of humidity at 38 °C for different time periods (1–4 weeks, red and blue curve Figure 7a,b) to simulate environmental conditions and compared with the original material (black curve).

**Figure 7:**
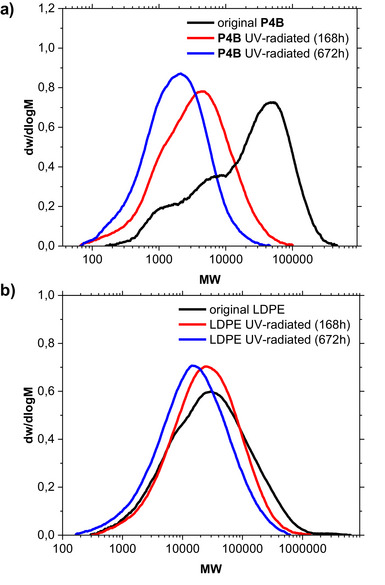
a) Molecular weight distributions of **P4B** before (black curve) and after the UV radiation (red and blue curve). b) Molecular weight distributions of commercial LDPE before (black) and after UV irradiation (red and blue curve).

We observed an accelerated decomposition of **P4B** compared to commercial LDPE. The NMR investigation revealed that this degradation process occurs preferentially on the carbonyl units of the ester groups (Figure S51).^[^ ^32^ ^]^ Moreover, the ester linkages can be cleaved under basic conditions to regain **P1** (Figures S28, S33).

In conclusion, we introduced the synthesis of multiply branched and long‐chain branched polyolefin material. It contains two types of functional groups: olefins and esters. The olefin‐type functional groups permit depolymerization and repolymerization and the ester groups allow a faster degradability or depolymerization. Our synthesis is efficient, highly flexible, and proceeds under mild conditions. Our material matches properties such as the melting point and tensile and rheological behavior of LDPE and is structurally related to LDPE.

## Supporting Information

Experimental details are given in the Supporting Information. The authors have cited additional references within the Supporting Information.

## Conflict of Interests

The authors declare no conflict of interest.

## Supporting information



Supporting Information

## Data Availability

The data that support the findings of this study are available in the supplementary material of this article.
