# Supplementary material for: A Multiply and Long‐Chain Branched Polyolefin with Low Density Polyethylene (LDPE)‐Like Properties Containing Two Types of Functional Groups
Source: Angew Chem Int Ed Engl. 2025 Oct 23;64(51):e202518150. doi: 10.1002/anie.202518150 (PMC12707347; doi:10.1002/anie.202518150)
Supplement: Supplementary file 1 — Supporting Information [file ANIE-64-e202518150-s001.docx]

Supplementary Material for

**A multiply and long-chain branched polyolefin with LDPE-like properties containing two types of functional groups**

C. Unger, C. Heber, H. Schmalz, C. Bauer, W. P. Kretschmer and R. Kempe

Table of Contents

[Materials and Methods 3](#_Toc200610022)

[Synthetic Procedures 6](#_Toc200610023)

[NMR Spectra 13](#_Toc200610024)

[GPC 26](#_Toc200610025)

[DSC 29](#_Toc200610026)

[TGA 31](#_Toc200610027)

[Tensile Tests 32](#_Toc200610028)

[Rheology 39](#_Toc200610029)

[UV-Degradation 40](#_Toc200610030)

[GC 43](#_Toc200610031)

[Literature 44](#_Toc200610032)

# Materials and Methods

General Considerations

All manipulations of air and/or moisture sensitive compounds were performed under exclusion of oxygen by using standard Schlenk line techniques or an argon or nitrogen filled glove box (mBraun) with a high-capacity circulator (< 0.1 ppm O_2_).

Chemicals

Toluene (Sigma Aldrich, anhydrous, 99.8 %) and ethylene (3.0, Linde AG) used for (co)polymerization experiments were passed over columns of R3-11 supported Cu oxygen scavenger (BASF) and Al_2_O_3_ (Fischer Scientific). Triethyl aluminum (AlEt_3_, TEA) (SASOL Germany GmbH), tetra-*n*-butylammonium bromide (Alfa), HCl (Kraft), sodium hydroxide pellets (Sigma Aldrich), Bicyclo[2.2.1]hept-5-ene-2-carboxylic acid (BLD pharm), N,N-Dimethylformamid (Acros), Oxalyl chloride (TCI), Dichloromethane (Fisher Chemical), Hoveyda-Grubbs catalyst 2^nd^ generation (C_31_H_38_Cl_2_N_2_ORu) (BLD pharm), cis-Cyclo-octene (Acros), Ethylvinylether (EVE) (Acros) and ethanol *p*.*a*. (Fisher Chemical) were used without further purification. 1‑Hexene (Acros) was degassed, stirred over LiAlH_4_ for 12 hours at room temperature, transferred under vacuum and stored under argon prior to usage. The *d*‑MAO (depleted) was obtained by removing the volatile components from a methylaluminoxane solution in toluene (Chemtura Europe Limited). Commercial Lupolen LDPE 1800P was purchased from LyondellBasell.

Characterization Methods

*^1^H and ^13^C NMR* - Spectra were performed at a Varian INOVA 300 (^1^H: 299.9 MHz, ^13^C: 75.4 MHz). The ^1^H- and ^13^C-NMR polymer spectra were recorded at 120 °C in C_2_D_2_Cl_4_ or at 80 °C in C_6_D_6_ and referenced internally to the residual solvent resonances (C_2_D_2_Cl_4_: ^1^H-NMR: 5.90 ppm; ^13^C‑NMR: 74.12 ppm, C_6_D_6_: ^1^H-NMR: 7.16 ppm; ^13^C‑NMR: 128.1 ppm). The ^1^H-NMR spectra of the low molecular weight compounds were recorded at 25 °C in C_6_D_6_ and referenced also internally to the residual solvent resonances. For the ^13^C-NMR measurements 10 000 number of transitions and a relaxation delay of 9 s was used. Chemical shifts (δ) are reported in ppm.

*Gel Permeation Chromatography (GPC)* - Analyses were carried out at an Agilent (Polymer Laboratories Ltd.) PL-GPC 220 high temperature chromatographic unit equipped with refractive index and differential pressure detectors, a guard and three linear mixed bed columns (2 x AppliChrom, Olexis). HT-SEC analyses were performed at 150 °C using 1,2,4-trichlorobenzene as mobile phase. The samples (10 - 20 mg) were prepared by dissolving the polymer (0.1 wt.-%) in the mobile
phase, heating in an external oven and running them without further filtration.
The obtained molecular weights were referenced to linear narrow distributed polyethylene (HDPE) standards (Mw = 110 – 430000 g·mol-1; Mark-Houwink para-meters: K = 40.6, α = 0.725).

*Differential Scanning Calorimetry (DSC)* - DSC measurements were performed on a Mettler DSC 3+. Standard aluminum pans with a volume of 40 µl were used. The samples were measured in two cycles from 20 to 210 °C, a heating rate of 10 K/min and under a nitrogen atmosphere. The T_m_ was taken from the second heating/cooling curve.

*Thermogravimetric Analysis (TGA)* - All TGA measurements were performed on a Mettler Toledo TGA / SDTA 851e. Standard aluminum pans with a volume of 40 μL were used. Samples were measured from 20 °C to 700 °C with a heating rate of 10 K/min under a nitrogen atmosphere.

*Compression Molding* - Compression molding was conducted at a Carver Typ 2518 hotpress. Films around 0.5 mm thickness were compression molded at 130 °C or 140 °C for 4-5 minutes at a pressure of 1 ton. All polymer processing was performed without the addition of additives (stabilizers or antioxidants).

*Tensile Elongation Tests* – The tensile tests were performed on an Instron universal testing machine 5565, equipped with a video extensometer using dogbone-shaped samples [0.5 mm (T) x 4 mm (W) x 15 mm (L)]. Tensile testing was performed at a ramp speed of 10 mm/min or 5 mm/min. Four replicates per material were reported for the elongation to break tests. The test with the highest elongation to break was used for the shown figures. Tensile tests for commercial LDPE measured with a ramp speed of 10 mm/min were taken from previous report.^[2]^

*Accelerated Weathering (UV-Degradation)* – UV-Degradation studies were performed according to DIN-EN-ISO4892-2. Disc-like samples were placed into a test chamber Q‑SUN XE-3 (Q-LAB Corporation, Westlake, OH, USA) equipped with three xenon lamps and a common Daylight-Q filter and irradiated with 60 W/m^2^ (at 300 - 400 nm), corresponding to a total irradiance of 594 W/m^2^. Relative humidity was set to around 50%, and the chamber temperature to 38 °C. Compression molded samples (thickness = 1 mm, diameter = 15 mm) were radiated for 168 hours (1 week), or 672 hours (4 weeks) without the addition of antioxidants.

*Rheology-Experiments* – The rheology measurements were conducted on an Anton Paar MCR 302 compact rheometer in plate-plate geometry (diameter *D* = 25 mm). The rheometer was equipped with an electrical Peltier plate (P-ETD 400) and a Peltier hood (H-ETD 400) for temperature control. Sample discs (thickness = 1 mm, diameter = 25 mm) were previously molded on a Carver Typ 2518 hotpress at 140 °C for 6 minutes at a pressure of 1 ton. The polymer processing for synthesized polymer samples was performed with the addition of the commercially available *Irganox 1010* as stabilizer. All measurements were performed in the linear viscoelastic regime, being determined prior to each measurement via respective strain sweeps. Frequency sweeps (0.1 - 600 rad/s) in the melt were performed at (130 °C, 150 °C and 170 °C) using nitrogen as protective gas. Frequency sweeps for commercial LDPE were taken from previous publication.^[2]^

*Transmission Electron Microscopy (TEM)* - Transmission Electron Microscopy was carried out by using a JEOL JEM 2200FS (200 kV) device. For the sample preparation, the samples were suspended in chloroform and sonicated for 5 min. For analysis, LC200-Cu grids were used.

*Elemental Analysis & ICP-OES* - Elemental analysis were carried out on an Elementar UNICUBE® device. The platinum content was determined by Inductively Coupled Plasma Optical Emission Spectrometry (ICP-OES). The fusion of the catalysts was carried out in a CEM MARS 6 microwave, for the ICP-OES measurements a SPECTRO ARCOS (Spectro Ametek) was used.

# Synthetic Procedures

Synthesis of Bicyclo[2.2.1]hept-5-ene-2-carboxylic acid chloride:

The synthesis of the acid chloride was conducted similar to published procedures.^[1]^ 3 g of 5-norbornene-2-carboxylic acid (14.5 mmol, 1.00 eq) were dissolved in 20 mL dichloromethane under an inert gas atmosphere at 0°C. 8 drops of dimethylformamide are added. Subsequently, 3.68 g oxalyl chloride (29 mmol, 2.00 eq) is slowly added. The mixture is warmed to room temperature and stirred for 18 hours. All volatiles are removed under reduced pressure and the product is obtained as yellow oil.

Synthesis of N-SiCN:

For the preparation of the N-SiCN support material a published procedure was modified.^[4]^ The synthesis was conducted in independent batch mode experiments. 200 mg of the polysilazane “KiON HTT 1800” (Clariant Advanced Materials GmbH), 0.988 mL (0.800 g, 15.08 mmol) of acrylonitrile and 75 mg azobis-(isobutyronitrile) were dissolved in 4 mL dimethylformamide and crosslinked for 16 h at 75 °C. After solvent removal under reduced pressure, the obtained greenbody was pyrolyzed using the following program.

Figure S1: Synthesis procedure for N-SiCN.

After ball milling for 40 minutes, 500 mg of the ceramic were washed by stirring in a solution of 6.7 mL NaOH (1 M) and 5 mL MeOH at 90 °C for 24 h. Afterwards, the material was washed until neutrality and dried at room temperature.

Synthesis of Pt/N-SiCN:

The catalyst was synthesized to contain approximately 20 wt% platinum. The synthesis of the Pt/N-SiCN catalyst was performed by wet impregnation of Pt(acac)_2_ (403.24 mg) with N‑SiCN (1000 mg) in toluene (20 mL). The solvent was completely evaporated at 120 °C. Subsequent pyrolysis and reduction of the material were performed under nitrogen, forming gas (90/10 N_2_/H_2_) and synthetic air (80/20 N_2_/O_2_) atmosphere. An example pyrolysis program for pyrolysis and reduction is given below.

Figure S2: Synthesis procedure for the hydrogenation catalyst Pt/N-SiCN.

TEM analysis:


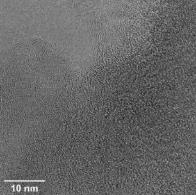

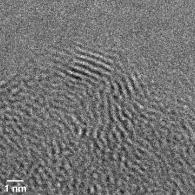

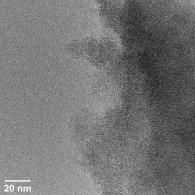

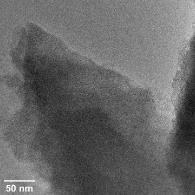

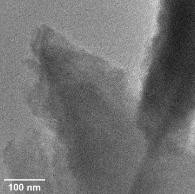

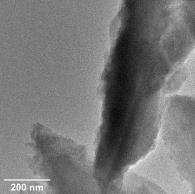


Figure S3:Survey transmission electron microscopy (TEM) of the N-SiCN support material as well as high resolution images. One can see graphitic domains within the N-SiCN support (red box) with a median domain size of 1.81 nm and a median interlayer distance of 3.66 Å.


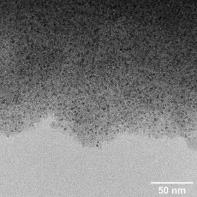

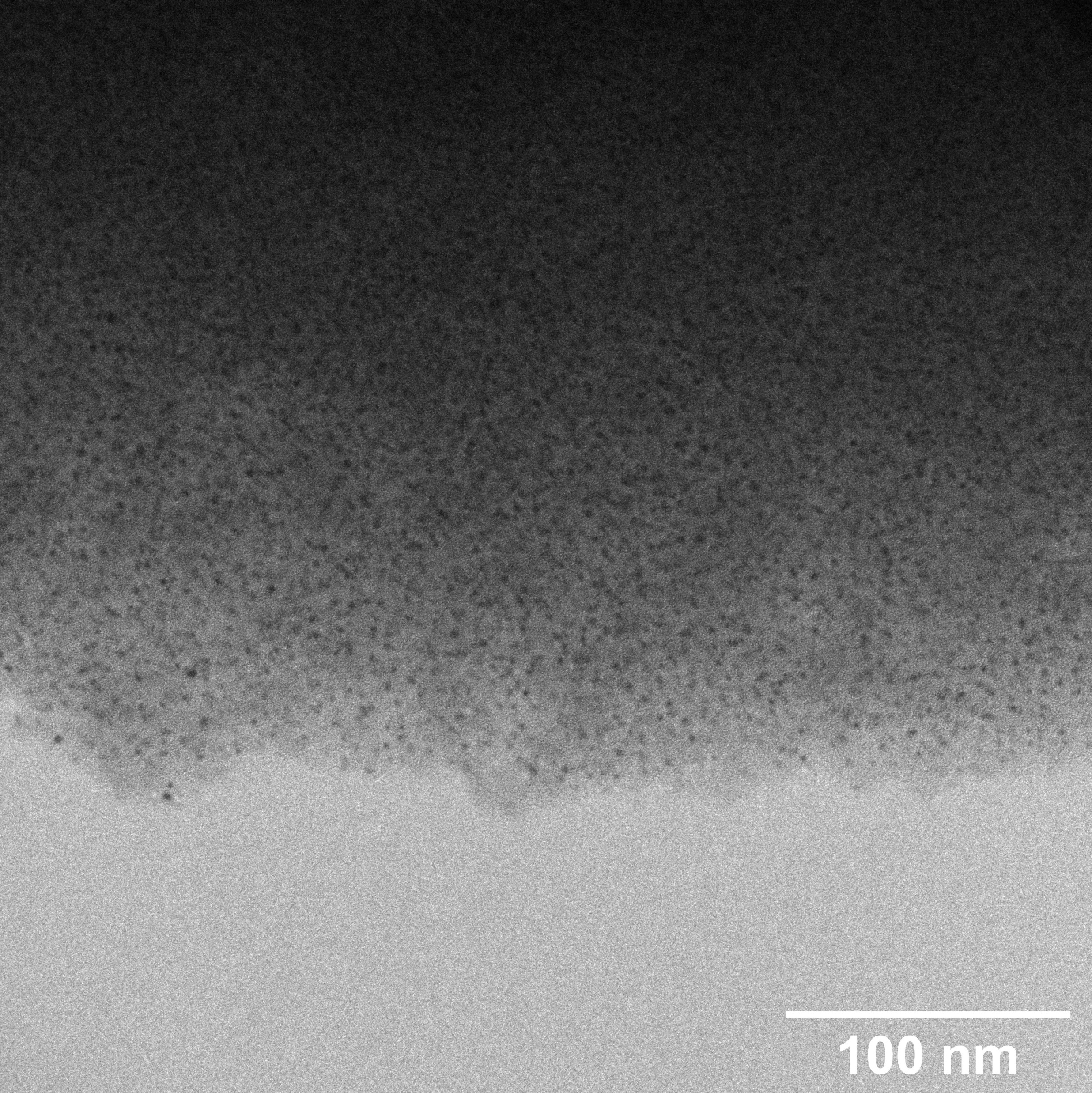

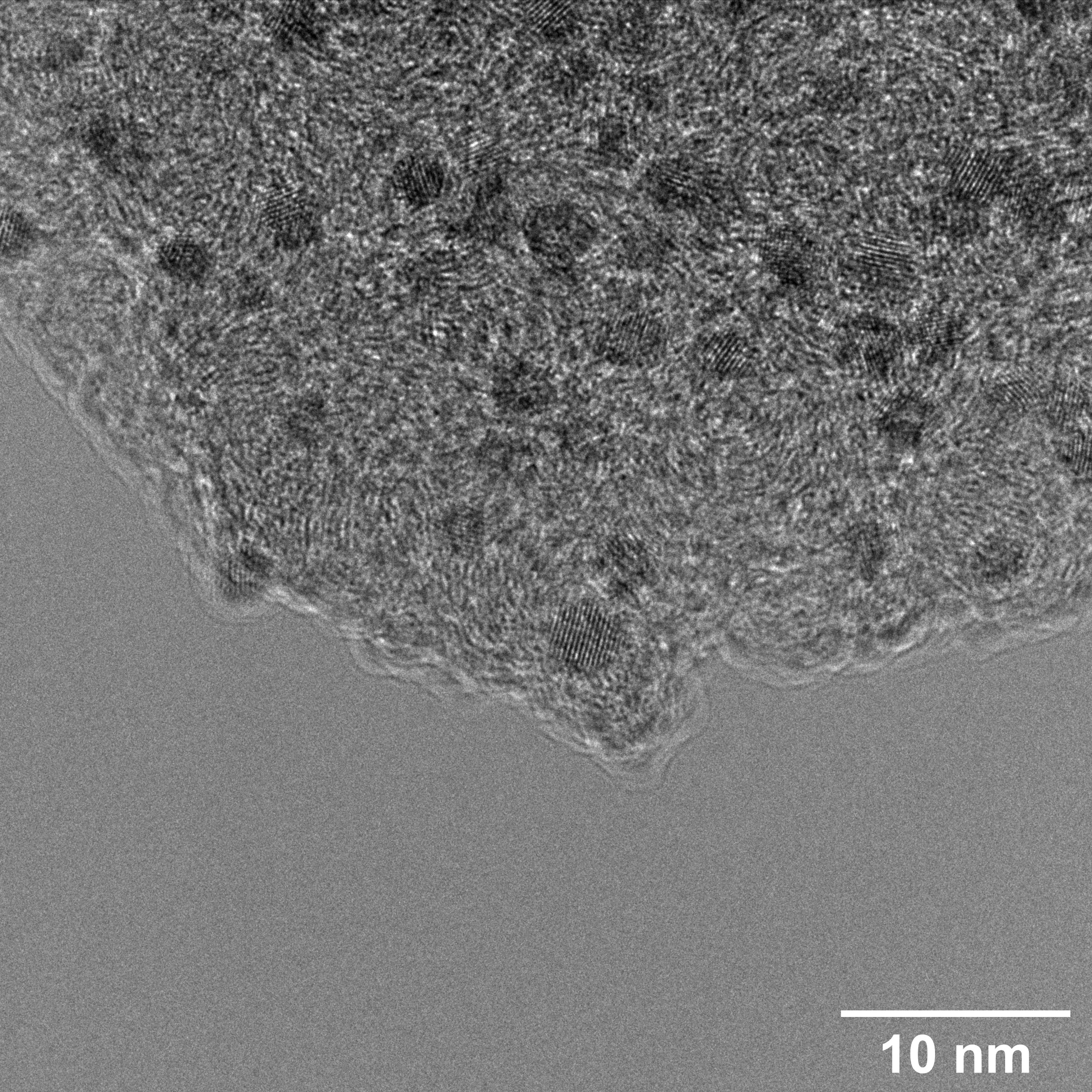


Figure S4: Survey recording of TEM images of the catalyst Pt/N-SiCN pyrolyzed at 700 °C and reduced at 550 °C. The platinum nanoparticles are homogeneously dispersed with a particle size distribution centred at 2.2 nm. High resolution TEM indicates the presence of defined nanoparticles.

Elemental Analysis:

Elemental analysis confirms the N-doping of the support material of the synthesized catalysts. The N-doping was calculated from the ratio of the nitrogen content to the carbon content.

Table EA: Elemental analysis of the support material and the catalyst materials.

| **Material** | **Pt [%]^[a]^** | **C [%]** | **H [%]** | **N [%]** | **N-doping C [%]** |
| --- | --- | --- | --- | --- | --- |
| Pt/N-SiCN | 18.8 | 67 | 0 | 6 | 9.0 |
| ^[a]^Platinum loading measured via ICP-OES. | | | | | |

Polymer synthesis:

The synthesis of all long chain alcohols **P1** ((co)polymerizations and oxidations) were conducted in a 1000 mL temperature and pressure controlled stainless steel autoclave with a mechanical stirrer (500-1000 rpm). All precatalysts, TEA, comonomers and activator were added as stock solutions in toluene. During the polymerization (semi batch mode) the ethylene pressure was kept constant by replenishing ethylene flow. The ethylene consumption was measured with a Bronkhorst High-Tech EI-Mass-Flow controller in liter normal (L_n_), 1 mol gas = 22.41 L_n_ at p = 1.01325 bar and 0 °C.

General synthesis of long chain alcohols **P1**:

The steel autoclave was evacuated (30 min) and heated to 80 °C. For the ethylene homopolymerization the autoclave was charged with 150 mL dry toluene, stirred at 1000 rpm, and pressured with ethylene (3 bar). TEA, *d*-MAO and 1 μmol precatalyst were subsequently added as stock solutions in toluene to reach total 180 mL solution. For the synthesis of the copolymer alcohols **P1** also 50 mL 4-EO were added, so that the reaction was conducted in 180 mL toluene/4-EO solution. 2 μmol of the precatalyst were used. After desired ethylene consumption ethylene was removed and pure oxygen (3 bar) was pressured to the autoclave and stirred at 80 °C and 1000 rpm for 3 hours. The reaction was quenched with ethanol. The polymer was precipitated from ethanol, washed with HCl/H_2_O, filtrated, washed with acetone, and dried in the oven (70 °C) under reduced pressure. The results are listed in Table S1.

**Table S1:** Data for the **P1** batch mode synthesis.

| Entry | TEA [mmol] | V_eth_ [L_n_] | 4-EO [mL] | M_n_ [g/mol] | M_p_ [g/mol] | $\text{Ð}$ |
| --- | --- | --- | --- | --- | --- | --- |
| 1^a)^ | 2.5 | 8 | / | 1 360 | 1 930 | 1.5 |
| 2 | 2.5 | 8 | 50 | 1 640 | 2 410 | 1.5 |
| 3 | 2.0 | 16 | / | 3 530 | 4 170 | 1.3 |
| 4 | 2.0 | 16 | 50 | 3 440 | 4 840 | 1.6 |

a) Data for Entry 1 Table S1 were taken from previous publication.^[2]^

General synthesis of the norbornene terminated macromonomer **P2**:

Figure S5: General synthesis of norbornene-terminated macromonomers.

In a Schlenk flask, 4.0 g **P1** (Table S1 Entry 4, ~1.3 mmol chains, 1.0 eq) were dissolved in 40 mL toluene and mixed with 306 mg Bicyclo[2.2.1]hept-5-ene-2-carboxylic acid chloride (1.95 mmol, 1.5 eq) and heated under an inertgas atmosphere to 130 °C. The reaction was stirred for 18 h and the polymer was precipitated by adding ethanol. The product (white powder) was dried under reduced pressure. The results of the P2 batch mode synthesis are listed in Table S2.

**Table S2:** GPC data of the **P2** batch mode synthesis (used long chain alcohols **P1** left, synthesized macromonomers **P2** right).

| **Entry*** | **M_n_(P1) [g/mol]** | $\text{Ð}$ **of P1** | **M_n_(P2) [g/mol]** | **M_p_(P2) [g/mol]** | $\text{Ð}$ **of P2** |
| --- | --- | --- | --- | --- | --- |
| **5** | 1 640 | 1.5 | 2 000 | 2 470 | 1.4 |
| **6** | 3 440 | 1.6 | 3 690 | 5180 | 1.6 |

General Synthesis of the unsaturated copolymers **P3**:

Figure S6: General Synthesis of P3.

In a Schlenk flask, 500 mg **P2** were tried to dissolve in 30 mL toluene at 45 °C - 55 °C under an argon atmosphere. Cis-Cyclooctene (COE) was mixed with toluene and added (either directly or step wise) to the slurry solution. The HG-II catalyst, dissolved in toluene, was added directly. The solution was stirred for 4 hours and stopped by the addition of ethylvinylether (EVE) (1 mL). The polymer was precipitated out of ethanol, washed with acetone and dried under reduced pressure. The results of the batch mode synthesis of **P3** are listed in Table S3.

**Table S3:** Data of the **P3** batch mode synthesis.

| **Entry** | **COE [g]** | **P2 [g]** | **P2 [Type]** | **M_n_ [g/mol]** | **M_p_ [g/mol]** | $\text{Ð}$ | **Addition time of the COE [min]** |
| --- | --- | --- | --- | --- | --- | --- | --- |
| **7** | 2.0 | / | / | 670 | 810 | 1.5 | direct |
| **8** | 2.0 | 0.5 | A | 1 520 | 3 000 | 2.8 | direct |
| **9A** | 2.0 | 0.5 | B | 2 670 | 7 280 | 3.6 | direct |
| **9B** | 2.0 | 0.5 | B | 1 400 | 3 440 | 4.5 | direct |
| **10** | 2.0 | 0.5 | B | 1 360 | 3 560 | 3.8 | 30 |
| **11** | 2.0 | 0.5 | B | 2 070 | 5 740 | 3.9 | 180 |

General Hydrogenation Reactions:

In a glas flask, 2.0 g of **P3** were mixed with 50 mg Pt/N-SiCN and 40 mL dry toluene. The flask was transferred to a stainless-steel autoclave. The autoclave was rinsed three times with hydrogen. The autoclave was pressured with hydrogen (30-40 bar) and heated to 100 °C. The solution was stirred for 15-18 h. The hydrogen pressure was released, the solid was decanted from the solution and the polymer was precipitated out of ethanol. The polymer was filtrated, washed with acetone and dried under reduced pressure. For purification the polymer was dissolved again and the solution decanted from the solid. The results of the **P4** batch mode synthesis are listed in Table S4.

**Table S4:** Synthesis conditions, GPC data and melting point of the **P4** batch mode synthesis.

| **Entry** | **Used P3** | **p (H_2_) [bar]** | **Hydrogenation time [h]** | **Degree of hydrogenation [%]** | **M_n_ [g/mol]** | **M_p_ [g/mol]** |  | $\text{Ð}$ | **T_m_ [°C]** |
| --- | --- | --- | --- | --- | --- | --- | --- | --- | --- |
| **12** | Entry 10 | 40 | 18 | 85 | 5 000 | 51 860 |  | 8.0 | 115 |
| **13** | Entry 11 | 40 | 18 | 74 | 5 700 | 36 140 |  | 6.8 | 103 |
| **14** | Entry 9B | 30 | 15 | 79 | 5 500 | 41 840 |  | 7.1 | 110 |

Depolymerisation of **P4** by ethenolysis:

In a 100 mL steel autoclave 1.7 g of **P4** (Entry 12, Table S4) were dissolved in 50 mL toluene, heated up to 80 °C and stirred for 1 hour. HG-II catalyst (3.4 mg, ~0.2 wt %) was dissolved in 3 mL toluene. 1 mL of this solution was added and 10 bar of ethylene were pressured to the autoclave. The rest of the catalyst solution (2mL) was added stepwise over 3 hours. The ethylene pressure was released, the reaction was stopped by adding 1 mL of EVE, and the polymer was precipitated out of ethanol, washed with acetone and dried under reduced pressure. The depolymerized product **P5** was yielded as powder.

Repolymerisation of **P5** by acyclic diene metathesis polymerization (ADMET):

750 mg of **P5** were diluted in a mixture of 5 mL toluene and 25 mL *n*-hexane at 95 °C HG-II catalyst (3.7 mg, ~0.5 wt%) were diluted in 3 mL toluene. 1 mL of the catalyst solution were added, and the mixture was stirred for 1 hour under an inert gas atmosphere at 95 °C (reflux conditions). 1 mL of the catalyst solution was added, and the reaction was stirred for 1 hour at 95 °C. Another 1 mL of the catalyst solution was added, and the solution was stirred for 4 hours. The reaction was stopped by adding EVE 1 mL, the polymer was precipitated out of ethanol, washed with acetone and dried under reduced pressure.

Saponification of **P4**:

In a pressure tube 200 mg of **P4** were mixed with 10 mg tetra-n-butyl-ammonium bromide and 1 mL NaOH (4N). 4 mL of toluene are added, and the solution is heated up to 100 °C. The solution is stirred for 22 hours at 100 °C, cooled to room temperature. The polymer is precipitated out of ethanol and washed with acetone. The polymer is dried under reduced pressure.

# NMR Spectra


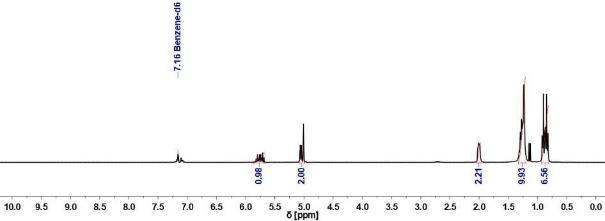


**5**

**4**

**4,5**

**3**

**3**

**2**

**2**

**1**

**1**

Figure S7: ^1^H-NMR-Spectrum of 4-EO.


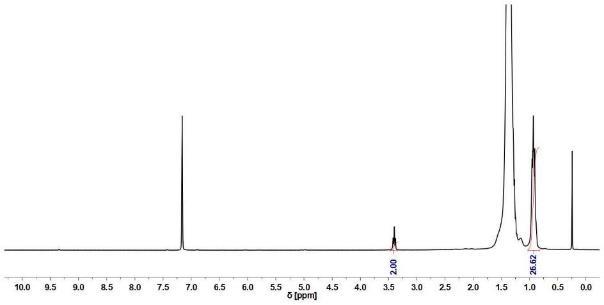


**4**

**3**

**2**

**1**

**2,3,4**

**11**

Figure S8: ^1^H-NMR-Spectrum of LLDPE-OH (Entry 2, Table S1).


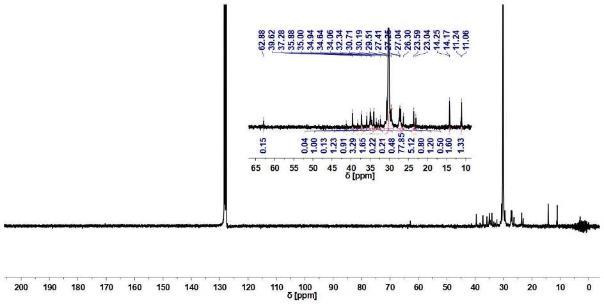

$$\text{α}$$

**^2^E_2_**

**^1^E_2_**

**^6^H_6_**

**^5^H_6_**

**^4^H_6_**

**^3^H_6_**

**^2^H_6_**

**^1^H_6_**

**^2^H_6_**

**^1^H_6_**

$$\text{β}$$

**1e**

**2e**

**1**

**1**

Figure S9: ^13^C-NMR-Spectrum of LLDPE-OH (Entry 2, Table S1).

Calculation of incorporated 4-EO-units per chain:

As an example, the calculation of average incorporated 4-EO units per single polymer chain is conducted for the copolymer alcohol of Table S1, Entry 2. For this calculation, the average polymer chain length M_n_ of the corresponding GPC data and the ^13^C-NMR spectrum of the 4‑EO/ethylene copolymer alcohol (Figure S7) are used.

**Table** **S5:** ^13^C-NMR data of the copolymer alcohols **P1** of Figure S7.

| $\text{δ}$ **[ppm]** | **assignment** | **name of the integral** |
| --- | --- | --- |
| **10-45** | Ethylene **C1** + Ethylene **C2**  + **^1^H_6_**-**^6^B_6_** + **^1^E_2_** + **^2^E_2_** + **α** + **β** | **I_Total_** |
| **14-14.5 + 23-23.6** | (**^1^H_6_** + **^2^H_6_**)·1/2 | **I_4EO_** |

The incorporation of the monomers in mol% is determined from the average mean molecular weight M_n_ and the integral values from Table S5. An integral value for ethylene (I_Eth_) is calculated from the integral values for 4-EO (I_4EO_) and the total integral in the range between 10 ppm and 45 ppm (I_Total_).

I_Eth_ = (I_Total_ - 10·I_4EO_)·1/2

X_4EO_ = $\frac{\text{I}_{\text{4EO}}}{\text{ }\text{I}_{\text{Eth}}\text{+ }\text{I}_{\text{4EO}}}$ $\text{≈}$ 3,9 mol %

X_Eth_ = $\frac{\text{I}_{\text{Eth}}}{\text{ }\text{I}_{\text{Eth}}\text{+ }\text{I}_{\text{4EO}}}$ $\text{≈}$ 96,1 mol %

To calculate the average 4-EO units per chain, the incorporation of the single monomers (mol %) and M_n_ is used. The average number of carbon atoms per chain N_C_ is determined.

N_C_ = M_n_ / 14 g/mol

Afterwards, the total number of carbons C_total_ is determined by using the calculated mol% for ethylene and 4-EO units and their carbon count.

C_total_ = 10X_4EO_ + 2X_Eth_

The average number of ethylene-units A_Eth_, and 4EO-units A_4EO_ per single polymer chain is determined as follows:

A_4EO_ = X_4EO_ · (N_C_/C_total_)

A_Eth_ = X_Eth_ · (N_C_/C_total_)

The results for the calculation of incorporation of the ethylene/4-EO copolymer alcohols (Entry 2 and 4 of Table S1) are listed in Table S6.

**Table S6**: Calculation of average monomer units per polymer chain for **P1**.

| used batch of Table S1 | Mn [g/mol] | $\text{Ð}$ | A_4EO_ | A_Eth_ |
| --- | --- | --- | --- | --- |
| Entry 2 | 1 640 | 1,5 | 2.0 | 48.7 |
| Entry 4 | 3 440 | 1,6 | 3.2 | 106.9 |

Calculation of the degree of hydroxy functionalization after oxidation: To determine the degree of hydroxy functionalization for the chain length of the ethylene (4-EO) (co)polymers the ^1^H-NMR spectrum for the PE-OH homopolymer (Figure S8, Entry 3, Table S1) was used. The resonances of the protons next to the hydroxy functionalization C*H*_2_-OH **1** were set to 2.00. The degree of functionalization **x** was determined by the following calculation:

I_Res(C_*_H_*_3)_ = I_2_ – 3.00

I_Res(norm)_ = I_Res(C_*_H_*_3)_ / 6

x = 1 / (1+I_Res(norm)_)

x = 1 / (1+0.42) = 0.703 ≈ 70 % (Entry3, Table S1)

The degree of hydroxy-functionalization for Entry1, Table S1 was taken from previous report (~74 %).^[2]^


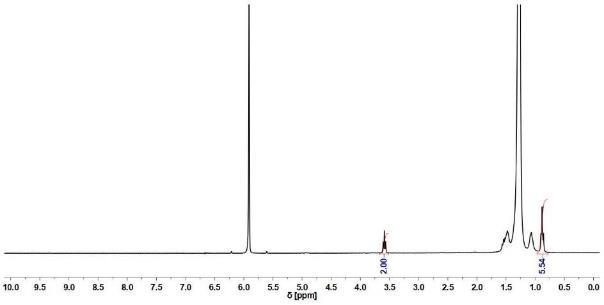


**1**

**1**

**2**

**2**

Figure S10: ^1^H-NMR-Spectrum of PE-OH (Entry 3, Table S1).


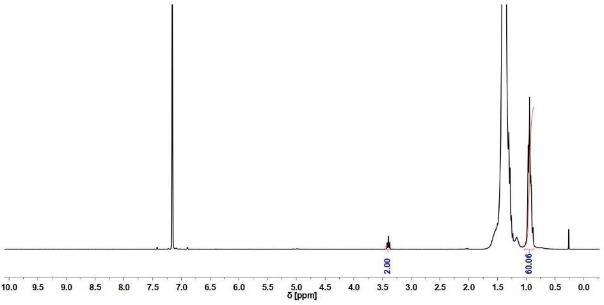


**4**

**3**

**2**

**1**

**2,3,4**

**1**

Figure S11: ^1^H-NMR-Spectrum of LLDPE-OH (Entry 4, Table S1).


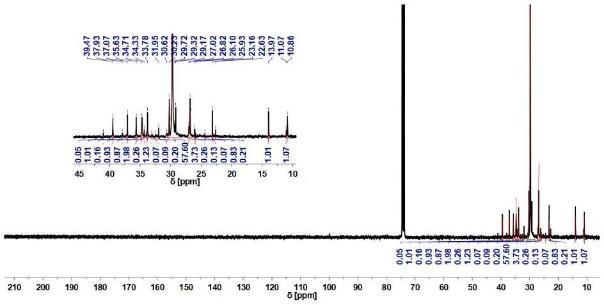


**^1^E_2_**

**^2^E_2_**

**^6^H_6_**

**^5^H_6_**

**^4^H_6_**

**^3^H_6_**

$$\text{α}$$

$$\text{β}$$

**2e**

**1e**

**1**

**^2^H_6_**

**^2^H_6_**

**^1^H_6_**

**^1^H_6_**

Figure S12:^13^C-NMR-Spectrum of LLDPE-OH (Entry 4, Table S1).


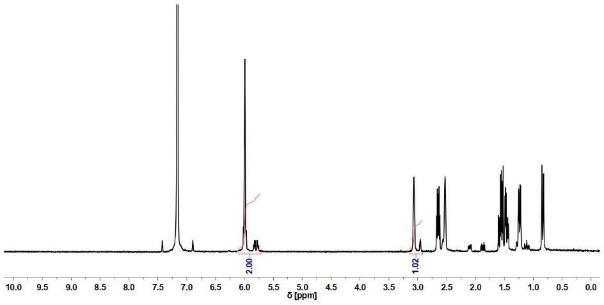


**3**

**3**

**1,2**

**2**

**1**

Figure S13: ^1^H-NMR-Spectrum of 5-norbornene-2-carboxylic acid.


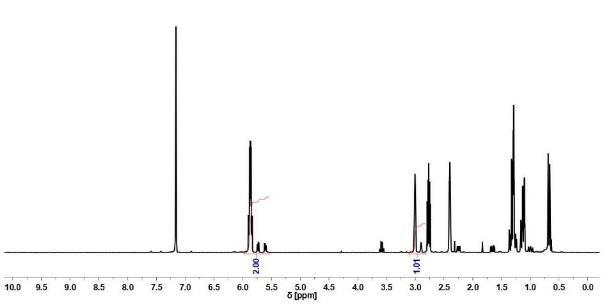


**3**

**3**

**2**

**1**

**1,2**

Figure S14: ^1^H-NMR-Spectrum of 5-norbornene-2-carboxylic acid chloride.


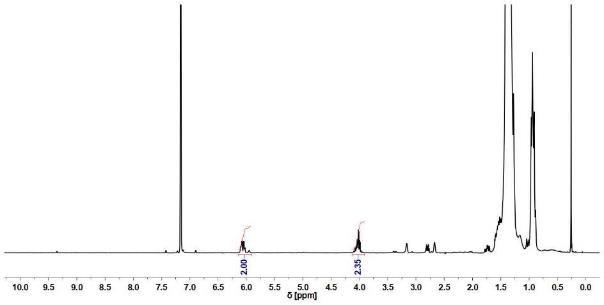


**3**

**1,2**

**3**

**2**

**1**

Figure S15: ^1^H-NMR-Spectrum of NB-LLDPE (Entry 5, Table S2).


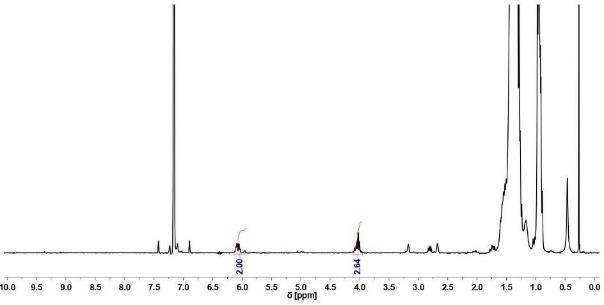


**1**

**2**

**3**

**3**

**1,2**

Figure S16: ^1^H-NMR-Spectrum of NB-LLDPE (Entry 6, Table S2).


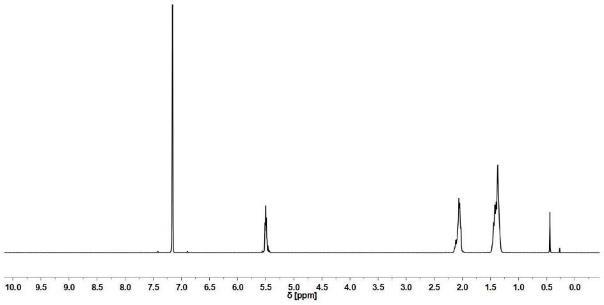


**3**

**2**

**1**

**4**

**3,4**

**1**

**2**

Figure S17: ^1^H-NMR-Spectrum of PCOE (Entry 7, Table S3).


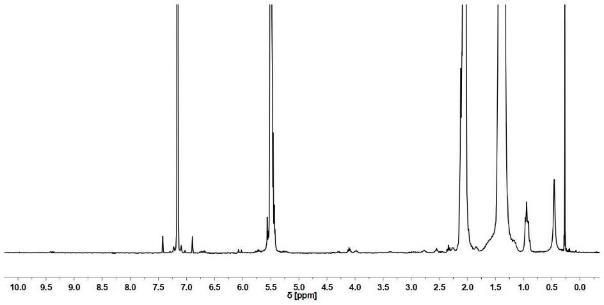


**1**

**4**

**3**

**5**

**2**

**5**

**1-4**

Figure S18: ^1^H-NMR-Spectrum of P(COE-g-LLDPE) (Entry 8, Table S3).


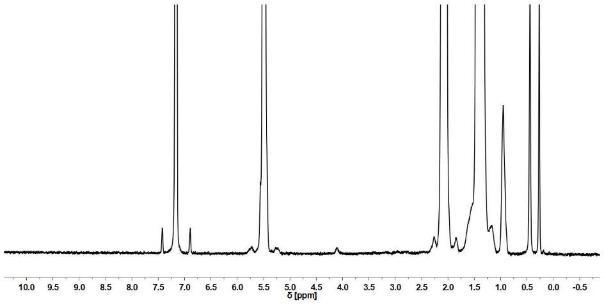


**5**

**4**

**3**

**1**

**2**

**1-4**

**5**

Figure S19: ^1^H-NMR-Spectrum of P(COE-g-LLDPE) (Entry 9A, Table S3).


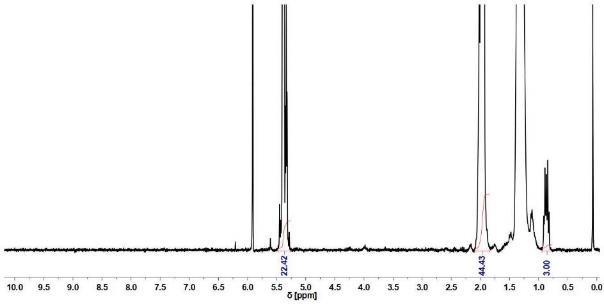


**3**

**2**

**5**

**1-4**

**5**

**4**

**1**

Figure S20: ^1^H-NMR-Spectrum of P(COE-g-LLDPE) (Entry 9B, Table S3).


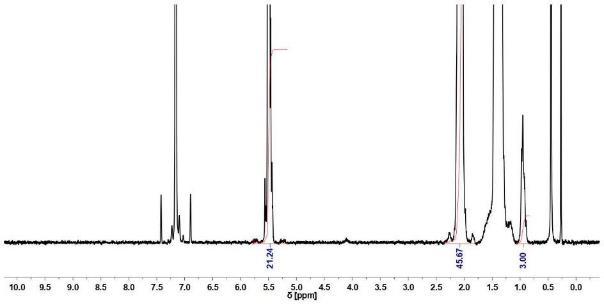


**5**

**4**

**2**

**3**

**1**

**5**

**1-4**

Figure S21: ^1^H-NMR-Spectrum of P(COE-g-LLDPE) (Entry 10, Table S3).


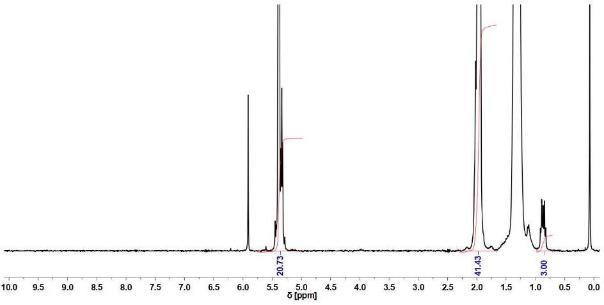


**5**

**3**

**2**

**1**

**4**

**5**

**1-4**

Figure S22: ^1^H-NMR-Spectrum of P(COE-g-LLDPE) (Entry 11, Table S3).


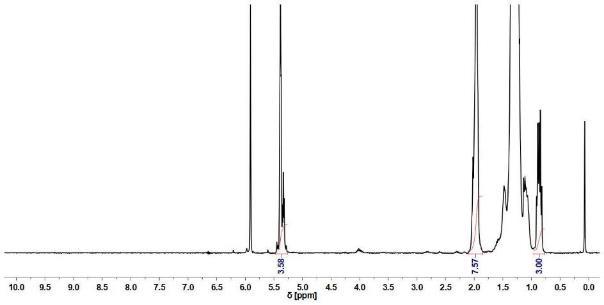


**1**

**2**

**3**

**3**

**1,2**

Figure S23: ^1^H-NMR-Spectrum of LCBPO (Entry 12, Table S4).


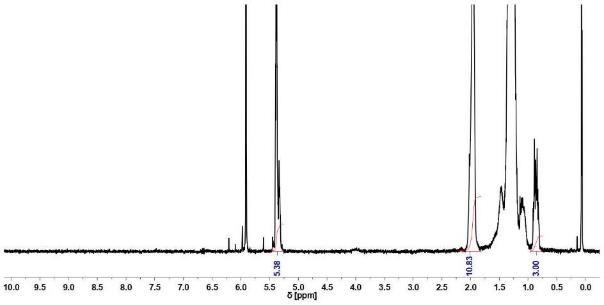


**1**

**2**

**3**

**3**

**1,2**

Figure S24: ^1^H-NMR-Spectrum of LCBPO (Entry 13, Table S4).


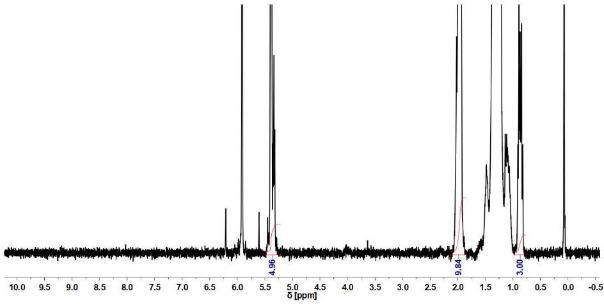


**1**

**2**

**3**

**3**

**1,2**

Figure S25: ^1^H-NMR-Spectrum of LCBPO (Entry 14, Table S4).


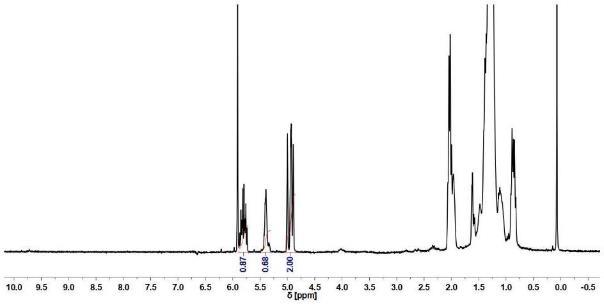


**4**

**5**

**6**

**2**

**1**

**3**

**7**

**7**

**5,6**

**2,3**

**1,4**

Figure S26: ^1^H-NMR-Spectrum of the depolymerized **P4** (depolymerized Entry 12, Table S4) by ethenolysis.


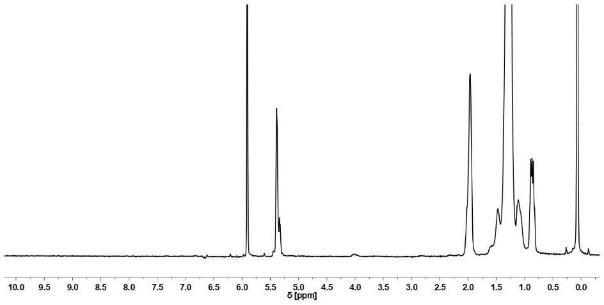


**4**

**1**

**5**

**2**

**3**

**5**

**1-4**

Figure S27: ^1^H-NMR-Spectrum of the repolymerized **P4** (repolymerized Entry 12, Table S4) by acyclic diene metathesis polymerization.


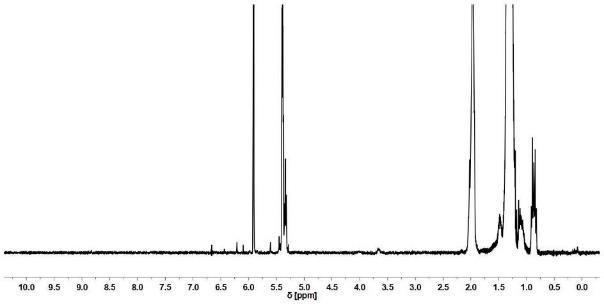


**1**

**2**

**3**

**3**

**1,2**

Figure S28: ^1^H-NMR-Spectrum of the depolymerized **P4** by basic saponification.

# GPC

Figure S29: Molecular weight distributions of the long chain alcohols **P1** (Table S1). The molecular weight distribution of Entry 1 was taken from previous report.^[2]^

Figure S30: Molecular weight distributions of the norbornene-functionalized macromonomers **P2** (Table S2).

Figure S31: Molecular weight distributions of P(COE-g-LLDPE) **P3** (Table S3).

Figure S32: Molecular weight distributions of **P4** (Entry 12-14, Table S4).

Figure S33: Molecular weight distribution of the degrafted **P4** (degrafted Entry 14).

Figure S34: Molecular weight distribution of commercial LDPE. Data was taken from previous report.^[2]^

# DSC

Figure S35: DSC (second heating curve) of the long chain alcohols **P1** (Table S1).

Figure S36: DSC (second heating curve) of P(COE-g-LLDPE) **P3** (Table S2).

Figure S37: DSC (second heating curve) of P(COE-g-LLDPE) **P3** (Table S3).

Figure S38: DSC (second heating curve) of LCBPO **P4** (Table S4) and commercial LDPE (taken from previous report).^[2]^

# TGA


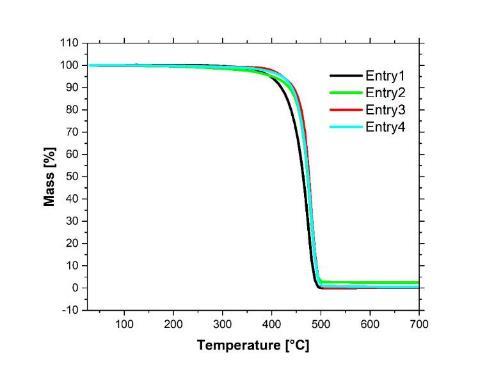


Figure S39: TGA curves of the long chain alcohols **P1** (Table S1).

Figure S40: TGA curves of **P3** (Table S3) and commercial LDPE (taken from previous report).^[2]^

# Tensile Tests


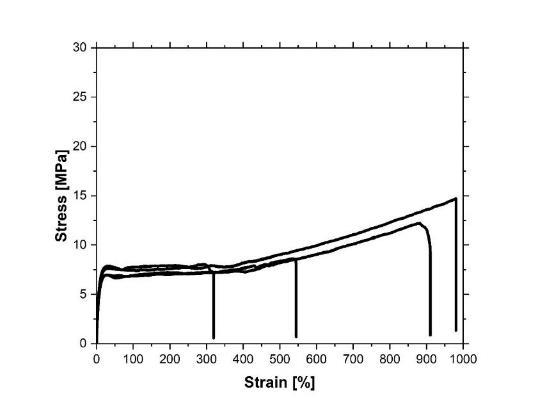


Figure S41: Tensile tests of PCOE (Entry 7, Table S3) measured with a ramp speed of 10 mm/min.

**Table S7:** Tensile tests of PCOE.

| Entry | Stress at Breaking Point [MPa] | Strain at Breaking Point [%] | Young´s Modulus [GPa] |
| --- | --- | --- | --- |
| 1 | 14.7 | 980 | 0.11 |
| 2 | 12.2 | 880 | 0.14 |
| 3 | 8.6 | 540 | 0.11 |
| 4 | 7.9 | 300 | 0.09 |
| average | **10.9** | **675** | **0.11** |
| standard deviation | **2.8** | **270** | **0.02** |


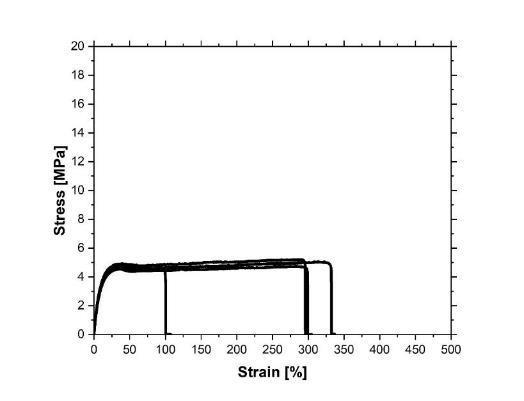


Figure S42: Tensile tests of **P3** (Entry 8, Table S3) measured with a ramp speed of 10 mm/min.

**Table S8:** Tensile tests of **P3** (Entry 8).

| entry | Stress at Breaking Point [MPa] | Strain at Breaking Point [%] | Young´s Modulus [GPa] |
| --- | --- | --- | --- |
| 1 | 4.9 | 330 | 0.02 |
| 2 | 5.2 | 290 | 0.06 |
| 3 | 4.5 | 300 | 0.05 |
| 4 | 4.4 | 100 | 0.04 |
| average | **4.8** | **255** | **0.04** |
| standard deviation | **0.3** | **90** | **0.01** |


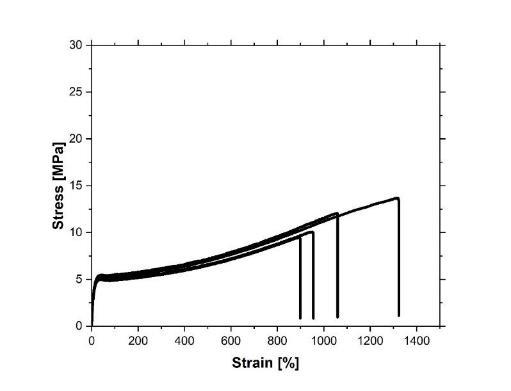


Figure S43: Tensile tests of **P3** (Entry 9A, Table S3) measured with a ramp speed of 10 mm/min.

**Table S9:** Tensile tests of **P3** (Entry 9A).

| entry | Stress at Breaking Point [MPa] | Strain at Breaking Point [%] | Young´s Modulus [GPa] |
| --- | --- | --- | --- |
| 1 | 13.7 | 1 320 | 0.08 |
| 2 | 12.1 | 1 060 | 0.05 |
| 3 | 10.0 | 950 | 0.06 |
| 4 | 9.4 | 900 | 0.07 |
| average | **11.3** | **1 060** | **0.07** |
| standard deviation | **1.7** | **160** | **0,01** |


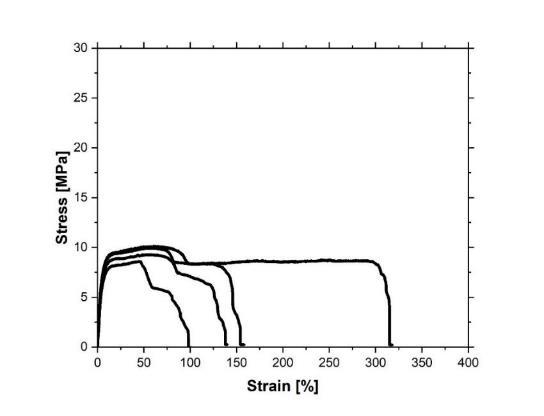


Figure S44: Tensile tests of **P4** (Entry 12, Table S4) measured with a ramp speed of 5 mm/min.

**Table S10:** Tensile tests of **P4** (Entry 12).

| entry | Stress at Breaking Point [MPa] | Strain at Breaking Point [%] | Young´s Modulus [GPa] |
| --- | --- | --- | --- |
| 1 | 8.5 | 300 | 0.28 |
| 2 | 7.9 | 135 | 0.28 |
| 3 | 6.3 | 120 | 0.29 |
| 4 | 5.5 | 80 | 0.24 |
| average | **7.1** | **160** | **0.27** |
| standard deviation | **1.2** | **85** | **0.02** |


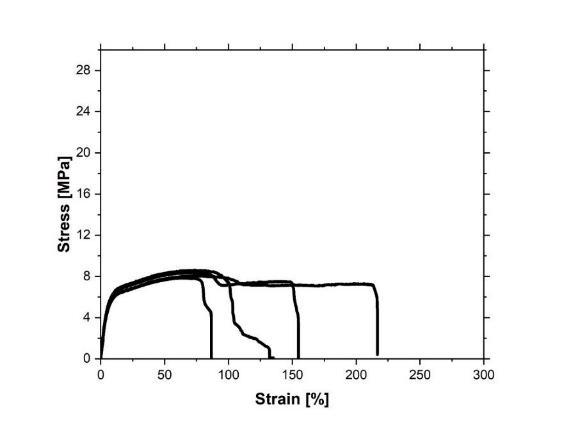


Figure S45: Tensile tests of **P4** (Entry 13, Table S4) measured with a ramp speed of 5 mm/min.

**Table S11:** Tensile tests of **P4** (Entry 13).

| entry | Stress at Breaking Point [MPa] | Strain at Breaking Point [%] | Young´s Modulus [GPa] |
| --- | --- | --- | --- |
| 1 | 7.1 | 210 | 0.18 |
| 2 | 7.3 | 150 | 0.21 |
| 3 | 7.2 | 100 | 0.14 |
| 4 | 7.5 | 80 | 0.19 |
| average | **7.3** | **135** | **0.18** |
| standard deviation | **0.15** | **50** | **0.03** |


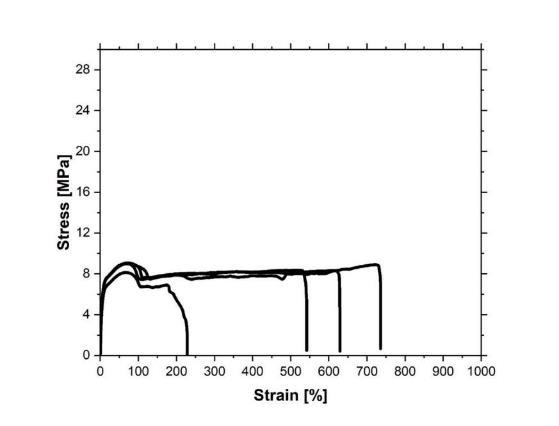


Figure S46: Tensile tests of **P4** (Entry 14, Table S4) measured with a ramp speed of 5 mm/min.

**Table S12:** Tensile tests of **P4** (Entry 14).

| entry | Stress at Breaking Point [MPa] | Strain at Breaking Point [%] | Young´s Modulus [GPa] |
| --- | --- | --- | --- |
| 1 | 6.8 | 180 | 0.18 |
| 2 | 8.8 | 730 | 0.19 |
| 3 | 8.2 | 630 | 0.19 |
| 4 | 8.3 | 530 | 0.21 |
| average | **8.0** | **520** | **0.19** |
| standard deviation | **0.74** | **210** | **0.01** |


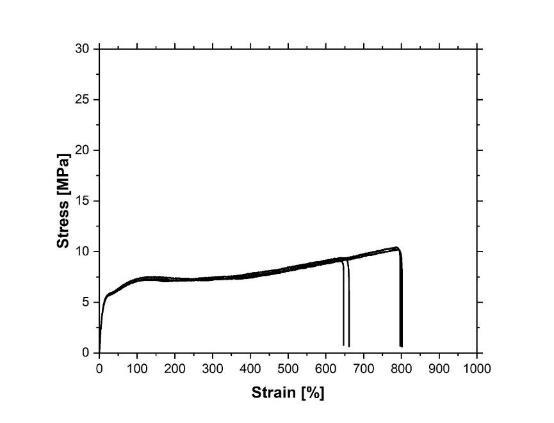


Figure S47: Tensile tests of commercial LDPE measured with a ramp speed of 5 mm/min.

**Table S13:** Tensile tests of commercial LDPE.

| entry | Stress at Breaking Point [MPa] | Strain at Breaking Point [%] | Young´s Modulus [GPa] |
| --- | --- | --- | --- |
| 1 | 10.1 | 790 | 0.1 |
| 2 | 10.4 | 790 | 0.1 |
| 3 | 9.4 | 650 | 0.1 |
| 4 | 8.9 | 640 | 0.1 |
| average | **9.7** | **720** | **0.1** |
| standard deviation | **0.6** | **70** | **0** |

# Rheology

Figure S48: Frequency sweep in the melt of **P4** (Entry 14, Table S4) at 150 °C.

Figure S49: Frequency sweep in the melt of **P4** (Entry 14, Table S4) at 170 °C.

# UV-Degradation

Figure S50: LCBPO before (left) and after (right) for weeks of irradiation by UV-Light.


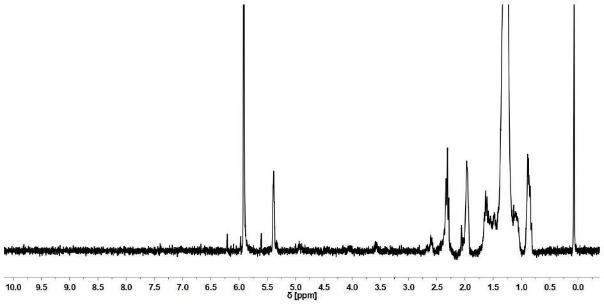


**2,3**

**2,3**

**3**

**2**

**1**

**2,3**

**1**

Figure S51: ^1^H-NMR analysis of the radiated **P4** (Entry 14, Table S4) (4 weeks).

Figure S52: Molecular weight distribution of **P4** (Entry 14, Table S4) UV-radiated for 168 h (1 week).

Figure S53: Molecular weight distribution of **P4** (Entry 14, Table S4) UV-radiated for 672 h (4 weeks).

Figure S54: Molecular weight distribution of commercial LDPE UV-radiated for 168 h (1 week).

Figure S55: Molecular weight distribution of commercial LDPE UV-radiated for 168 h (4 weeks).

# GC

Figure S56: GC-Spectra of distillated 4-EO used for copolymerisation experiments.

The monomer 4-EO was synthesized according to published procedure by elongation and branching of 1-hexene with two molecules of ethylene.^[3]^ The Product was distillated from co-products and used with a purity ­~92.4 % (determined by GC).

# Literature

[1] M. Leinert, T. Irrgang, R. Kempe *ChemCatChem.* **2023**, *15*, e202301042.

[2] C. Unger, H. Schmalz, J. Lipp, W. P. Kretschmer, R. Kempe, *Adv. Sci.* **2024**, 2307229.

[3] T. Dietel, F. Lukas, W. P. Kretschmer, R. Kempe, *Science* **2022**, *375*, 1021.

[4] L. F. B. Ribeiro, O. Flores, P. Furtat, C. Gervais, R. Kempe, R. A. F. Machado, G. Motz, *J. Mater. Chem. A* **2017**, *5*, 720–729.
